# A comparison of host response strategies to distinguish bacterial and viral infection

**DOI:** 10.1371/journal.pone.0261385

**Published:** 2021-12-14

**Authors:** Melissa Ross, Ricardo Henao, Thomas W. Burke, Emily R. Ko, Micah T. McClain, Geoffrey S. Ginsburg, Christopher W. Woods, Ephraim L. Tsalik

**Affiliations:** 1 Duke University School of Medicine, Durham, NC, United States of America; 2 Duke Center for Applied Genomics & Precision Medicine, Duke University School of Medicine, Durham, NC, United States of America; 3 Department of Electrical and Computer Engineering, Duke University, Durham, NC, United States of America; 4 Duke Regional Hospital, Durham, NC, United States of America; 5 Medical Service, Durham Veterans Affairs Health Care System, Durham, NC, United States of America; 6 Emergency Medicine Service, Durham Veterans Affairs Health Care System, Durham, NC, United States of America; Fundacao Oswaldo Cruz, BRAZIL

## Abstract

**Objectives:**

Compare three host response strategies to distinguish bacterial and viral etiologies of acute respiratory illness (ARI).

**Methods:**

In this observational cohort study, procalcitonin, a 3-protein panel (CRP, IP-10, TRAIL), and a host gene expression mRNA panel were measured in 286 subjects with ARI from four emergency departments. Multinomial logistic regression and leave-one-out cross validation were used to evaluate the protein and mRNA tests.

**Results:**

The mRNA panel performed better than alternative strategies to identify bacterial infection: AUC 0.93 vs. 0.83 for the protein panel and 0.84 for procalcitonin (P<0.02 for each comparison). This corresponded to a sensitivity and specificity of 92% and 83% for the mRNA panel, 81% and 73% for the protein panel, and 68% and 87% for procalcitonin, respectively. A model utilizing all three strategies was the same as mRNA alone. For the diagnosis of viral infection, the AUC was 0.93 for mRNA and 0.84 for the protein panel (p<0.05). This corresponded to a sensitivity and specificity of 89% and 82% for the mRNA panel, and 85% and 62% for the protein panel, respectively.

**Conclusions:**

A gene expression signature was the most accurate host response strategy for classifying subjects with bacterial, viral, or non-infectious ARI.

## Introduction

Emerging antibiotic resistance is one of the most pressing medical challenges of our time; in 2019 the Centers for Disease Control and Prevention estimated 2.8 million antibiotic resistant infections causing more than 35,000 deaths annually in the United States [1]. One contributing factor is a high rate of inappropriate antimicrobial use. Even among patients with upper respiratory tract infections, which are typically viral, antibacterials are prescribed in up to 42% of cases [2]. This is itself driven by difficulties identifying whether an illness is infectious and if so, whether it is bacterial or viral. To augment pathogen detection approaches, which have limitations, there has been growing interest in host response strategies. The best-studied clinically available biomarker to discriminate bacterial and non-bacterial etiologies of acute respiratory illness (ARI) is procalcitonin [3–6]. Previous studies have shown that despite being a relatively poor biomarker for distinguishing bacterial versus viral infection [7,8], procalcitonin-guided algorithms can reduce antibacterial consumption, decrease antibacterial-related side effects, and lower mortality [9,10].

Combining multiple host biomarkers in a multivariate index assay may offer better opportunities to discriminate bacterial and viral infections. Multi-protein biomarker panels have been developed [11,12]. Among them is a 3-peptide panel of IP-10 (interferon-inducible protein 10), TRAIL (TNF-related apoptosis-inducing ligand), and CRP (C-reactive protein), which has been shown to accurately distinguish bacterial ARI from viral ARI [12–14]. However, these studies did not include sufficient numbers of patients with acute respiratory illness of non-infectious etiology to gauge performance characteristics in a clinically relevant population. Moreover, subjects with equivocal test results were excluded from analysis, which inflates performance characteristics.

Host gene expression offers another diagnostic biomarker strategy for bacterial vs. viral infection [15–19]. Furthermore, patients with non-infectious illness can also be distinguished using host gene expression [18]. We have previously adapted this signature onto the BioFire FilmArray System, which is a rapid, sample-to-answer platform measuring host gene expression [20,21]. Validated in a large cohort of patients with ARI, this gene expression test accurately discriminated patients with bacterial infection, viral infection, coinfection, or no infection [22,23].

These biomarker strategies have been variably evaluated, precluding head-to-head comparisons. In this secondary analysis of the RADICAL study (Rapid Diagnostics in Categorizing Acute Lung Infection), we applied a uniform analytical strategy to directly compare the performance of multiple host response biomarkers including procalcitonin, a three-protein panel (IP-10, TRAIL, and CRP), and a 45-transcript mRNA panel. These biomarkers were evaluated in a cohort of 286 subjects with ARI of bacterial, viral, or non-infectious etiologies.

## Materials and methods

### Study cohort

Studies were approved by the Duke University Institutional Review Board (IRB), Durham VA Health Care System IRB, Henry Ford IRB, and University of North Carolina Medical Center IRB in accordance with institutional and federal regulations regarding human subjects’ protection. Written informed consent was obtained from all subjects or legally authorized representatives.

This study is reported as per the Strengthening the Reporting of Observational Studies in Epidemiology (STROBE) guideline (S1 Checklist). This is a secondary analysis of the RADICAL study, focusing on subjects with microbiologically confirmed bacterial ARI, viral ARI, or non-infectious illness and who had samples available to support all biomarker testing [23]. The study protocol and analysis plan for this study were not pre-specified at the time the RADICAL study was designed and implemented. Subjects with a low-confidence reference standard were excluded, consisting of those without confirmatory microbiology or bacterial/viral co-infection. Enrollment occurred in the emergency departments of Duke University Medical Center, Durham VA Health Care System, Henry Ford Hospital, and University of North Carolina Medical Center from 2005 through 2016. They were enrolled as part of CAPSOD (Community-Acquired Pneumonia and Sepsis Outcome Diagnostics; ClinicalTrials.gov NCT00258869) or CAPSS (Community-Acquired Pneumonia and Sepsis Study). Patients ≥6-years were eligible if they had a known or suspected infection and at least two Systemic Inflammatory Response Syndrome (SIRS) criteria [24]. Additional subjects were enrolled at Duke and the Durham VA as part of RADICAL (Rapid Diagnostics in Categorizing Acute Lung Infections). RADICAL enrolled subjects ≥2-years with <28-days duration of suspected bacterial, viral, or non-infectious ARI.

### Reference standard and case definitions

Since there is no gold standard to define whether ARI is bacterial, viral, or non-infectious, panel adjudication served as the reference standard [18,23,25,26]. Adjudications were performed by specialists in emergency medicine, infectious diseases, pulmonary medicine, or hospital medicine based on chart reviews performed >28 days after enrollment, and before measuring procalcitonin, the protein panel, or the mRNA panel. Due to the retrospective nature of the adjudications, the diagnostic accuracy of this reference standard is expectedly higher than what might be achieved in real-time by clinical providers. Adjudicators had access to medical records including but not limited to admission notes, consultant notes, discharge summaries, patient-reported symptoms, and laboratory and radiographic results (when clinically indicated). In addition, we performed supplemental microbiological testing including a multiplex viral respiratory pathogen panel (ResPlex V2.0, Qiagen; Respiratory Viral Panel, Luminex; or Respiratory Pathogen Panel, Luminex). Each case was independently adjudicated by two reviewers. If there was discordance with respect to the primary etiology, the case was reviewed by a panel of at least three reviewers. To be categorized as having a viral or bacterial ARI, a subject must have had a compatible clinical syndrome and an identified pathogen. The determination of “noninfectious illness” required an alternative non-infectious diagnosis and negative microbiological testing. Sepsis was defined based on the Sepsis-2 criteria, which requires the presence of systemic inflammatory response syndrome (SIRS) due to infection [24].

### Procalcitonin measurement

**Fig 1:**
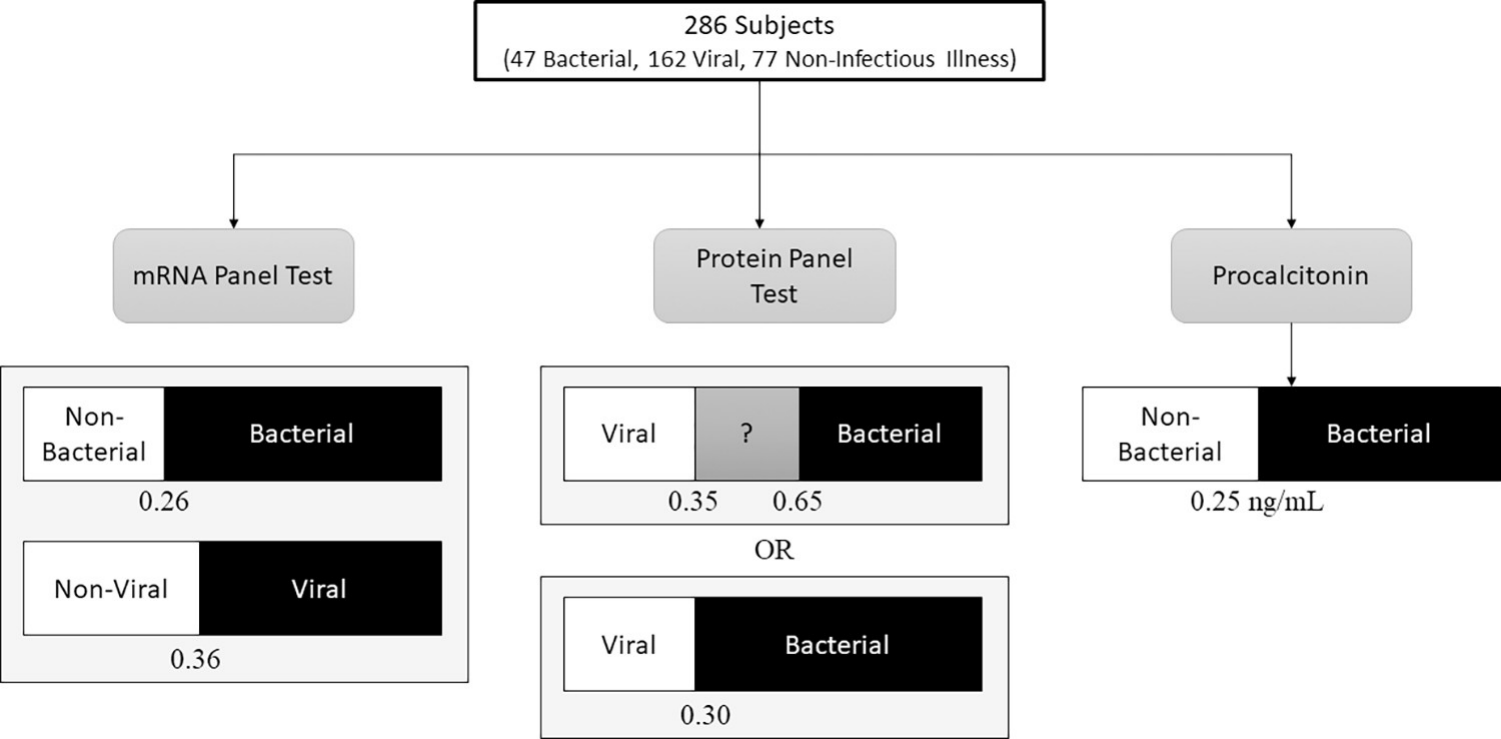
Study schematic. The analysis cohort included 286 subjects, each of which had three bacterial vs. viral biomarkers measured. The mRNA panel results were based on a previously published study using the BioFire system to measure 45 host response transcripts. This test reports independent results for bacterial vs. non-bacterial (viral or non-infectious illness) and viral vs. non-viral (bacterial or non-infectious illness), each with its own threshold. The protein panel test measures IP-10, TRAIL, and CRP. Two sets of thresholds were applied. The first, based on published values, assigns values below 0.35 as Viral, values above 0.65 as Bacterial, and values from 0.35–0.65 as equivocal. A second scheme was utilized whereby a single optimized threshold of 0.30 was used to distinguish bacterial from viral infections. The third biomarker, procalcitonin, used the established threshold of 0.25 ng/ml to distinguish bacterial from non-bacterial etiologies.

Procalcitonin was measured for study purposes and was not available to adjudicators, limiting any potential incorporation bias. Serum measurements were made on a Roche Elecsys 2010 analyzer (Roche Diagnostics) by electrochemiluminescence immunoassay or the miniVIDAS immunoassay (bioMérieux). Measurements in plasma-EDTA were made by the Phadia Immunology Reference Laboratory by immunofluorescence using the B·R·A·H·M·S PCT sensitive KRYPTOR (Thermo Fisher Scientific). Since all three platforms are approved for this indication, results were treated equivalently regardless of test platform. Subjects were classified as bacterial (≥0.25 ng/mL) or non-bacterial (<0.25 ng/mL) (Fig 1).

### Protein panel measurement

Concentrations of plasma IP-10, TRAIL, and CRP were measured by sandwich immunoassay with electrochemiluminescent detection using the UPLEX Human IP-10 Assay, the UPLEX Human TRAIL Assay, and the VPLEX Human CRP Assay with imaging using the QuickPlex SQ 120 Imager (Meso Scale Discovery, Rockville, MD). Plasma samples stored at -80°C were thawed on ice and processed according to the manufacturer’s recommended protocol, similar to published methods [12]. Calibration curves were generated using 4-fold serial dilutions of the calibrator. Generating probabilities of bacterial vs. viral infection was performed as previously described: protein concentrations were used to build a multinomial logistic regression model, establishing a bacterial likelihood score from zero to one [12].

Subjects were classified as bacterial or viral using two different thresholds (Fig 1). The first used published thresholds: subjects with a score ≤0.35 were classified as viral or non-infectious, subjects with a score ≥0.65 were classified as bacterial, and scores from 0.35–0.65 were considered equivocal [12]. The second method identified a single ideal threshold, above which subjects were classified as bacterial and below which subjects were classified as viral. We used the Youden Index to identify a threshold that maximized sensitivity and specificity. Values ≥0.30 were considered bacterial and values <0.30 were considered non-bacterial.

### mRNA panel measurement

The composition and development of the mRNA panel measured in this study was previously described [21]. In brief, the gene signature test is composed of two unique host response signatures: a bacterial host response signature that assigns a probability of having a bacterial vs. non-bacterial etiology and a viral host response signature that assigns a probability of having a viral vs. non-viral etiology (Fig 1). The two probabilities are independent and do not sum to 1, which allows for the identification of co-infection (i.e., both positive) or no infection (i.e., both negative). In order to generate these probabilities, the BioFire System measured transcript abundance, which was then used to build a logistic regression model trained on subjects with known phenotype.

Gene expression was measured via research-use-only FilmArray pouches as previously described [21]. At initial clinical presentation, subjects were enrolled and whole blood was collected directly into PAXgene Blood RNA tubes (PreAnalytix) and stored at -80°C until use. For testing, 100μL was used to measure the relative abundance of 45 target host mRNAs as defined by the PCR cycle at which the amplified target was detected using FilmArray chemistry and processing. Normalized target expression values were used to build two independent sparse logistic regression models: one to discriminate viral from non-viral etiologies and the other to discriminate bacterial from non-bacterial etiologies. The regularization parameter of the model and performance metrics were estimated using nested Leave-One-Out Cross-Validation (LOOCV), where the internal LOOCV was used for the regularization parameter and the outer LOOCV for performance estimates. This approach was selected to match the model training and validation process used for the protein panel. Thresholds for the bacterial (0.263) and viral (0.361) tests were chosen to optimize the average weighted accuracy [27].

### Statistical analysis

Each of the three tests was evaluated for its ability to distinguish bacterial from non-bacterial subjects. The protein panel and gene signature were also evaluated for their ability to distinguish viral from non-viral subjects. Procalcitonin was not evaluated for viral vs. non-viral discrimination since it does not discriminate these two possibilities. Further, a combined model was created using a multinomial logistic regression model, treating each of the aforementioned tests as an individual input. Test performance was evaluated by comparing the area under the receiver operating characteristic curve (AUC), sensitivity, specificity, positive predictive value (PPV), and negative predictive value (NPV). Biomarker strategies were compared using the Chi-squared test except for AUC, which was compared using the Delong test. Data is available in S1 Table.

## Results

### Demographics

Samples from 286 subjects were analyzed. Subjects had ARI due to clinically adjudicated bacterial (n = 47), viral (n = 162), or non-infectious (n = 77) etiologies. Demographics were similar across different disease states. Subjects with bacterial infection had a mean age of 52 years, were 58% male, 43% African American, and 2% Hispanic. Subjects with viral infection had a mean age of 43 years, were 40% male, 58% African American, and 4% Hispanic. Subjects with non-infectious illness had a mean age of 54 years, were 52% male, and 41% African American (Table 1). The most common bacterial pathogens were *Streptococcus pyogenes*, *Streptococcus pneumoniae*, and *Staphylococcus aureus* (Table 2). The most common viral pathogens were influenza A, rhinovirus/enterovirus, respiratory syncytial virus (RSV), and human metapneumovirus (Table 2). The most common non-infectious etiologies included asthma and congestive heart failure (Table 2).

**Table 1:** Subject demographics.

	Bacterial (n = 47)	Non-infectious Illness (n = 77)	Viral (n = 162)	Total (n = 286)
Age, mean (SD)	52.5 (22.1)	54.4 (16.9)	42.7 (16.6)	47.5 (18.5)
Sex, Male (%)	28 (58.3)	40 (52.6)	65 (40.1)	133 (46.5)
Race, n (%)				
Black	21 (44.7)	31 (40.3)	95 (58.6)	147 (51.4)
White	26 (55.3)	43 (55.8)	61 (37.7)	130 (45.5)
Other/Unknown	0	2 (2.6)	4 (2.5)	6 (2.1)
Comorbidities				
Chronic Lung Disease	20 (42.6)	35 (45.5)	39 (24.1)	94 (32.9)
Coronary Artery Disease	11 (23.4)	18 (23.4)	9 (5.6)	38 (13.3)
Diabetes	13 (27.7)	19 (24.7)	34 (21.0)	66 (23.1)
Heart Failure	4 (8.5)	17 (22.1)	4 (2.5)	25 (8.7)
Hypertension	28 (59.6)	39 (50.6)	66 (40.7)	133 (46.5)
Immunosuppressive Therapy	7 (14.9)	16 (20.8)	7 (4.3)	30 (10.5)
Malignancy	6 (12.8)	13 (16.9)	8 (4.9)	27 (9.4)
Hospitalized, n (%)	30 (63.8)	54 (70.1)	34 (21.0)	118 (41.3)

**Table 2:** Adjudicated phenotypes and etiologies.

Etiology	Number of Subjects
Bacterial Infection	
*Streptococcus pyogenes*	11
*Streptococcus pneumoniae*	9
*Staphylococcus aureus*	7
*Haemophilus influenzae*	2
*Legionella pneumophila*	2
*Pseudomonas aeruginosa*	2
Other^a^	14
Viral Infection	
Influenza A	78
RSV	12
Metapneumovirus	12
Rhinovirus	11
Enterovirus/Rhinovirus	11
Coxsackievirus/Echovirus	8
Coxsackievirus/Echovirus + Rhinovirus	8
Coronavirus (not SARS-CoV-2)	6
Parainfluenza	6
Influenza B	4
Adenovirus	4
Epstein Barr Virus	2
Non-Infectious Illness	
Asthma	15
Congestive Heart Failure	15
Interstitial Lung Disease	6
Pulmonary Embolism	6
Chronic Obstructive Pulmonary Disease	5
Malignancy	5
Allergic Rhinitis	3
Chest Pain	2
Diabetic Ketoacidosis	2
Myocardial Infarction	2
Other^b^	16

^a^Other bacterial etiologies included one instance of each of the following: Beta hemolytic Streptococcus (not Group A), *Enterococcus faecalis*, *Escherichia coli*, *Klebsiella pneumoniae*, *Pasteurella multocida*, *Pseudomonas aeruginosa* and A*lcaligenes xylosoxidans*, *Staphylococcus aureus* and *Haemophilus influenzae*, *Staphylococcus aureus* and *Pseudomonas aeruginosa*, *Staphylococcus hominis*, *Streptococcus agalactiae* and Coagulase negative Staphylococcus, *Streptococcus anginosus*, Streptococcus Group C, *Streptococcus pneumoniae* and *Staphylococcus aureus*, and Viridans Group Streptococcus.

^b^Other non-infectious etiologies included one instance of each of the following: acute allograft rejection, acute respiratory distress syndrome, anaphylaxis, hemidiaphragm paralysis, hemoptysis, intoxication, malignant pleural effusion, migraine, nephrolithiasis, post-infectious cough, post-operative pain, post-operative vocal cord paralysis, pulmonary hypertension, subglottic papilloma, syncope, tracheobronchomalacia.

### Procalcitonin

**Fig 2:**
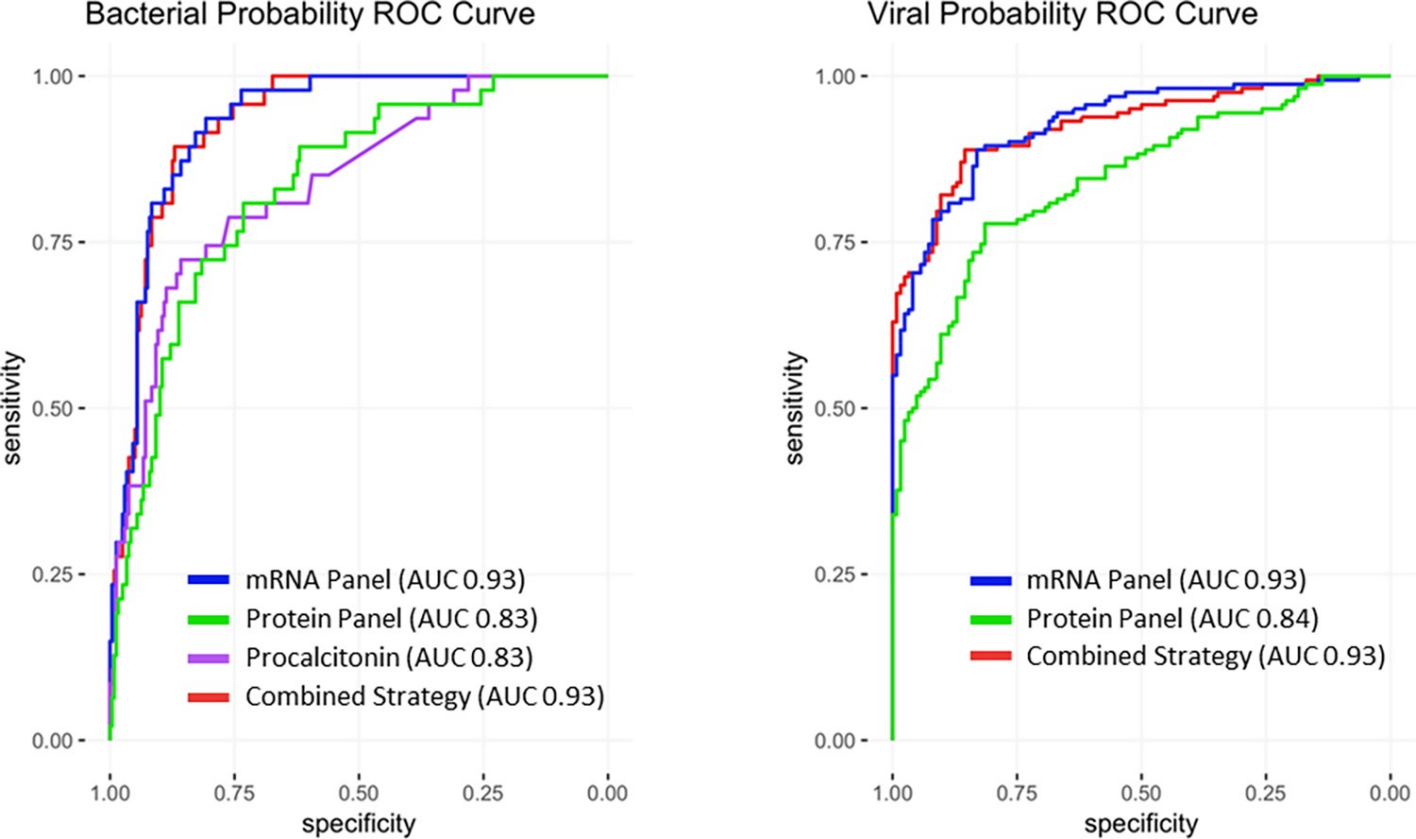
Bacterial and viral diagnosis receiver operating characteristic curves using different host biomarker strategies. Bacterial vs. non-bacterial classification is shown on the left. The combined strategy includes procalcitonin, protein panel, and mRNA panel. Viral vs. non-viral classification is shown on the right. For viral classification, the combined model includes the mRNA and protein panels.

Procalcitonin was used as a binary marker of bacterial infection, with a concentration ≥0.25 ng/ml denoting a bacterial infection and values <0.25 ng/ml indicating a non-bacterial process. In this cohort, procalcitonin differentiated bacterial from non-bacterial etiologies with 68.1% sensitivity (95% confidence interval [CI], 52.9%-80.9%), 86.6% specificity (95% CI, 81.6%-90.7%), and AUC of 0.837 (95% CI, 0.773 to 0.901) (Fig 2). These and additional test characteristics are shown in Table 3. Procalcitonin does not differentiate viral from non-infectious etiologies. As such, we did not apply it to the question of viral vs. non-viral (which includes bacterial and non-infectious etiologies).

**Table 3:** Test performance for bacterial vs. non-bacterial etiologies and viral vs. non-viral etiologies.

	AUC (95% CI)	Accuracy (95% CI)	Sensitivity (95% CI)	Specificity (95% CI)	PPV (95% CI)	NPV (95% CI)	LR+ (95% CI)	LR- (95% CI)
**BACTERIAL VS. NON-BACTERIAL ETIOLOGIES**								
**Procalcitonin**	0.837* (0.773, 0.901)	83.6% (78.8%, 87.7%)	68.1%* (52.9%, 80.9%)	86.6% (81.6%, 90.7%)	50.0% (40.7%, 59.3%)	93.2%* (90.1%, 95.5%)	5.09 (3.49, 7.42)	0.37 (0.24, 0.56)
**Protein Panel**	0.832* (0.771, 0.893)	74.5%* (69.0%, 79.4%)	80.9%* (66.7%, 90.9%)	73.2%* (67.1%, 78.7%)	37.3%* (31.6%, 43.4%)	95.1% (91.5%, 97.2%)	3.02 (2.35, 3.88)	0.26 (0.14, 0.47)
**mRNA Panel**	0.932 (0.902, 0.963)	84.3% (79.5%, 88.3%)	91.5% (79.6%, 97.6%)	82.9% (77.5%, 87.4%)	51.2% (43.9%, 58.4%)	98.0% (95.1%, 99.2%)	5.33 (3.98, 7.14)	0.1 (0.04, 0.26)
**Procalcitonin + Protein Panel + mRNA Panel**	0.931 (0.901, 0.962)	83.9% (79.1%, 88.0%)	91.5% (79.6%, 97.6%)	82.4% (77.0%, 87.0%)	50.6% (43.4%, 57.7%)	98.0% (95.1%, 99.2%)	5.21 (3.90, 6.94)	0.1 (0.04, 0.26)
**VIRAL VS. NON-VIRAL ETIOLOGIES**								
**Protein Panel**	0.843* (0.799, 0.888)	74.8%* (69.4%, 79.8%)	84.6% (78.1%, 93.3%)	62.1%* (53.0%, 70.7%)	74.5%* (69.7%, 78.7%)	75.5%* (67.7%, 81.9%)	2.23 (1.76, 2.82)	0.25 (0.17, 0.37)
**mRNA Panel**	0.928 (0.899, 0.956)	86.0% (81.5%, 89.8%)	88.9% (83.0%, 93.3%)	82.3% (74.4%, 88.5%)	86.8% (81.7%, 90.6%)	85.0% (78.4%, 89.8%)	5.01 (3.42, 7.35)	0.14 (0.09, 0.21)
**Protein Panel + mRNA Panel**	0.926 (0.896, 0.955)	87.4% (83.0%, 91.0%)	88.9% (83.0%, 93.3%)	85.5% (78.0%, 91.1%)	88.9% (83.9%, 92.5%)	85.5% (79.1%, 90.2%)	6.12 (3.98, 9.42)	0.13 (0.08, 020)

* p<0.05 compared to corresponding metric for the mRNA Panel. A procalcitonin concentration ≥0.25 ng/ml indicated bacterial infection. A protein panel probability ≥0.30 indicated bacterial infection. mRNA panel thresholds were ≥0.263 for bacterial classification and ≥0.361 for viral infection. CI = Confidence Interval, AUC = Area Under the Receiver Operating Characteristic Curve. PPV = Positive Predictive Value. NPV = Negative Predictive Value. LR+ = Positive likelihood ratio. LR- = Negative likelihood ratio.

### Protein panel test

#### Bacterial vs non-bacterial classification

**Fig 3:**
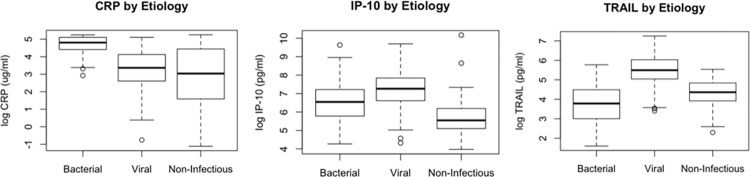
Log concentrations of CRP, TRAIL, and IP-10 stratified by clinical category. Data are presented as a box and whisker plot showing medians with interquartile ranges.

Consistent with their expected distributions, subjects with bacterial infections had higher concentrations of CRP and lower concentrations of TRAIL and IP-10 (Fig 3). Using previously published reporting recommendations, we classified results as bacterial (score ≥0.65), equivocal (score 0.35–0.65), or viral (score ≤0.35). Among 47 subjects with bacterial infection, 20 (43%) were classified as bacterial, 15 (32%) were equivocal, and 12 (25%) were viral. Of the 162 viral cases, 8 (5%) were classified as bacterial, 14 (9%) were equivocal, and 140 (86%) were correctly identified as viral. Finally, of the 77 subjects with non-infectious illness, 12 (16%) were classified as bacterial, 24 (31%) were equivocal, and 41 (53%) fell into the viral/non-infectious category. Overall, 19% of cases (n = 53) fell in the equivocal range and thus could not be classified. The overall accuracy accounting for all tested subjects using these previously published thresholds was 70.3% (95% CI, 64.6%-75.5%).

When equivocal results apply to such a large number of patients, generalizability becomes limited. We therefore used the previously established threshold of ≥0.65 to indicate the presence of a bacterial infection and any values <0.65 to indicate a non-bacterial etiology (viral infection or non-infectious illness). This resulted in a test sensitivity of 42.6% (95% CI 28.3%-57.8%) and specificity of 91.6% (95% CI 87.4%-94.8%). Since these protein panel thresholds were established in other clinical cohorts, they may not be well-calibrated to this study population. We therefore selected a new threshold that optimized test performance in this cohort. Using a threshold of 0.30, we observed a more balanced sensitivity [80.9% (95% CI, 66.7%-90.9%)] and specificity [73.2% (95% CI, 67.1%-78.7%)], which corresponded to an AUC of 0.832 (95% CI, 0.771–0.893) (Table 3, Fig 2).

#### Viral vs non-viral

Using the previously published threshold of ≤0.35, the protein panel had 86.4% sensitivity (95% CI, 80.2%-91.3%) but only 57.3% specificity (95% CI, 48.1%-66.1%) for the classification of viral infection. Most misclassifications were non-infectious subjects being classified as viral, which may have smaller clinical consequences as both groups would be spared antibacterial use.

Using the threshold optimized for this cohort described above (<0.30 for viral infection and ≥0.30 as non-viral), the protein panel had 84.6% sensitivity (95% CI, 78.1%-93.3%), 62.1% specificity (95% CI, 53.0%-70.7%), and AUC of 0.843 (95% CI, 0.799–0.888) for detecting viral infections (Table 3, Fig 2).

### mRNA panel test

#### Bacterial vs non-bacterial

**Fig 4:**
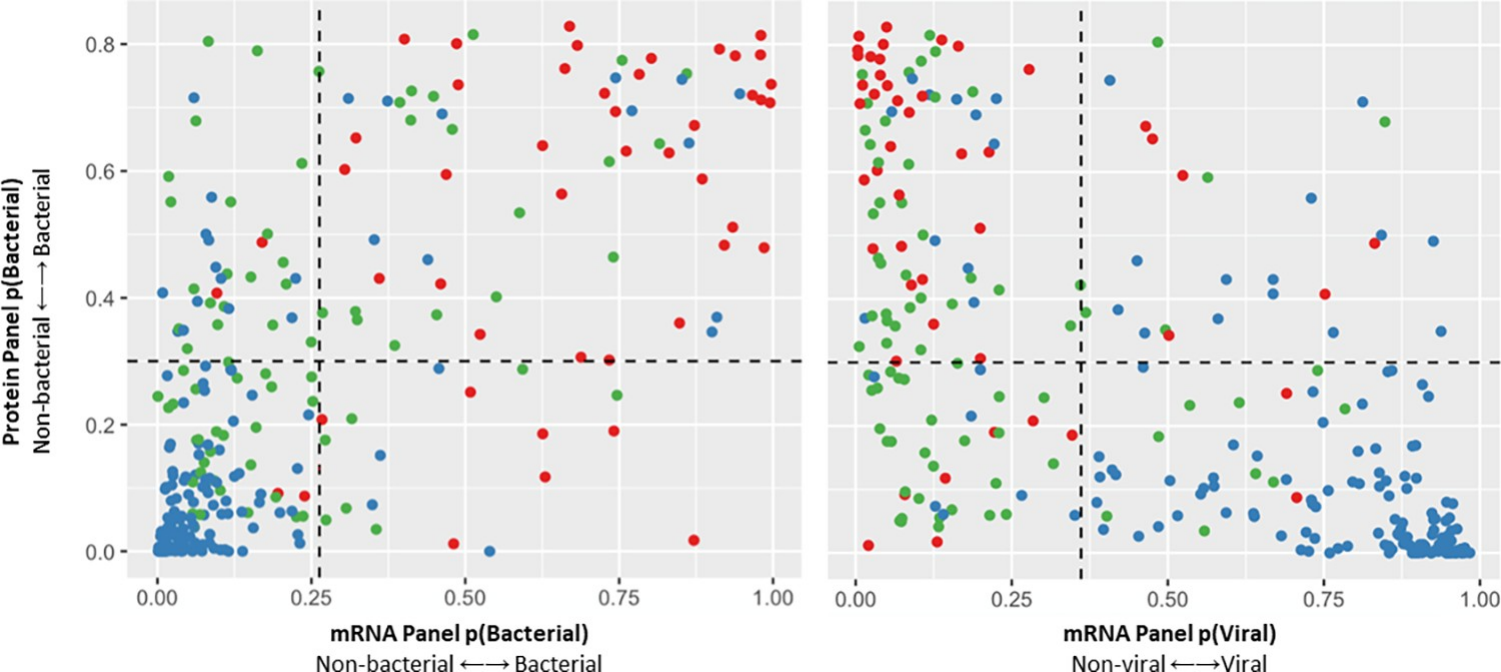
Distribution of predicted bacterial (left) and viral (right) infection probabilities using the protein and mRNA panels. The probabilities predicted by the protein panel test (y-axis) are plotted relative to the probabilities predicted by the mRNA panel test (x-axis). The dashed horizontal line corresponds to the optimal protein panel threshold. The vertical line corresponds to the optimal mRNA panel threshold. Bacterial infection cases are represented by red dots. Viral infection cases are represented by blue circles. Non-infectious illness cases are represented by green circles.

Any subject with a bacterial probability ≥0.26 using the mRNA panel test was classified as having a bacterial infection. Using this threshold, the mRNA panel test had 91.5% sensitivity (95% CI, 79.6%-97.6%), 82.9% specificity (95% CI, 77.5%-87.4%), and AUC of 0.932 (95% CI, 0.902–0.962) (Table 3, Fig 2). Specificity was significantly better than the protein panel (p <0.01), and sensitivity was significantly better than both procalcitonin and the protein panel (p<0.001) (Table 3). A direct comparison of the predicted probabilities of bacterial infection using the mRNA and protein panel tests is shown in Fig 4. A model combining the protein panel, mRNA panel, and procalcitonin did not improve performance beyond that of the mRNA panel alone (Table 3, Fig 2).

#### Viral vs non-viral

Any subject with a viral probability of ≥0.361 was classified as having a viral infection. Using this threshold, the gene signature distinguished viral vs. non-viral subjects with a sensitivity of 88.9% (95% CI, 83.0%-93.3%), specificity of 82.3% (95% CI, 74.4%-88.5%), and AUC of 0.928 (95% CI, 0.899–0.956) (Table 3, Fig 2). Specificity was significantly better than the protein panel (p <0.0001) (Table 3). A direct comparison of the predicted probabilities of viral infection using the mRNA and protein panel tests is shown in (Fig 4). Comparison to procalcitonin was not performed since it does not discriminate viral from non-infectious etiologies. A model combining the protein panel and mRNA panel did not improve performance beyond that of the mRNA panel alone (Table 3, Fig 2).

### Discordant classifications

We hypothesized there would be scenarios in which all three host response tests agreed with each other but disagreed with clinical adjudication. This occurred in nine cases. The only pattern we could discern among them were three cases adjudicated as having Group A Streptococcal pharyngitis, but all three host response tests identified a viral infection. In the entire cohort, there were 11 cases of Group A Streptococcal pharyngitis corresponding to a 27% (3/11) error rate.

We also stratified the cohort by the presence or absence of SIRS criteria, which in the case of bacterial and viral infections, would denote sepsis. SIRS was present in 155 subjects and absent in 131. We observed no differences in the sensitivity for any biomarker strategy, though this was limited by having only six bacterial infections without sepsis. In contrast, the specificity to diagnose a bacterial infection was lower for all three biomarker strategies among subjects with SIRS compared to those without: 78.1% vs. 94.4% for procalcitonin (p<0.001), 62.3% vs. 83.2% for the protein panel (p<0.001), and 71.9% vs. 92.8% for the mRNA panel (p<0.001).

There were no other identifiable patterns with respect to illness or pathogens among the other discordant predictions. Similarly, there was no clear pattern among cases where the different biomarker panels were discordant with each other.

## Discussion

ARI is among the most common acute care presentations worldwide. This syndrome is commonly due to viral, bacterial, or non-infectious etiologies. The greatest challenge in clinical management is identifying when a bacterial infection is present and subsequently, which patients need antibacterial therapy. Pathogen-detection strategies are an important component of this evaluation but have limited utility. A complementary strategy measures the host’s molecular response to the infection, which can discriminate between etiologies. This study evaluated three different host-based diagnostic approaches in a common cohort of patients with bacterial, viral, and non-infectious ARI. We showed that all three strategies discriminate between bacterial and viral etiologies, although an mRNA panel test performed best, in part because of its ability to discriminate patients with non-infectious etiologies.

Currently, procalcitonin is the most widely used biomarker to aid in the differentiation of bacterial and non-bacterial etiologies of ARI. Procalcitonin has been cleared by the FDA to guide the management of antibiotic therapy in patients with lower respiratory tract infection based on previously published trial data [28]. However, a recent study failed to show an impact by procalcitonin on antibacterial use, the reasons for which are likely multifactorial [29]. Other recent publications have demonstrated a poor ability to discriminate bacterial and viral etiologies [29,30]. Further, high false positive rates for procalcitonin have been observed for patients with a variety of inflammatory and chronic disease conditions [31–38]. The limitations of procalcitonin may be inherent to the use of a single biomarker approach. Consequently, integrating multiple biomarkers from different biological pathways into a single test could improve ARI diagnostics.

One example is the use of CRP, IP-10, and TRAIL, which were identified in a proteomic study based on their ability to differentiate bacterial and viral infections [12]. This panel has been validated in several studies, demonstrating an ability to distinguish bacterial from viral infection with a sensitivity of 86%-94% and specificity of 90%-94% [13,14,39,40]. Equivocal test results were obtained in 11–17% of these cohorts compared to 19% in this study. Such a high rate of non-informative results limits the utility of such a test. Furthermore, these prior publications excluded subjects with equivocal results when calculating sensitivity and specificity. Removing subjects from the denominator (all subjects tested who had a valid test result) will artificially inflate performance estimates. When performance characteristics are calculated with all tested subjects accounted for, results are expectedly lower. This was observed in our study as well as another independent validation performed by van der Does et al [41]. In that study, sensitivity was 78% and specificity was 73% for the diagnosis of bacterial infection, which is similar to our observations (81% and 73%, respectively).

Transcriptomic approaches present another opportunity for disease classification. Many transcriptomic-derived classifiers for distinguishing between patients with bacterial or viral infections have been described [15–19,42–50]. Despite these discoveries, the means to measure these signatures in a clinically meaningful time was not previously available. To address that, we translated the gene expression signature using BioFire technology, taking advantage of the sample-to-answer format [23]. Using this research-use-only test, the mRNA panel test evaluated here was better than the protein panel and procalcitonin for classifying subjects with ARI. One potential reason is that a 45-gene signature captures more biology due to the increased number of analytes measured. Perhaps similar improvements could be achieved with a larger panel of proteins although adding procalcitonin to the protein panel did not improve results. Furthermore, protein biomarkers for measurement in serum or plasma will be restricted to those that are secreted, representing a biologically limited view of the underlying host response. Furthermore, the mRNA panel was discovered in a cohort of patients that had a higher proportion of non-infectious etiologies as compared to the alternative biomarker strategies. As such, it is expected to perform better in an undifferentiated and more heterogeneous patient population.

This study identified Group A streptococcal pharyngitis as one diagnostically challenging area. Specifically, all three biomarker strategies were discordant with clinical adjudication in 27% of subjects with streptococcal pharyngitis. Rates of pharyngeal *S*. *pyogenes* colonization are as high as 40% depending on how the carrier state is defined [51]. This suggests that the presence of Group A Streptococcus in and of itself is a poor indication of the presence of bacterial infection. Host response biomarkers may therefore offer additional and perhaps superior diagnostic information for such infections.

More broadly, all three biomarker strategies offered high NPVs for bacterial infection, ranging from 93% for procalcitonin to 98% for the mRNA test. These values highlight the potential utility for such biomarkers in mitigating inappropriate antibacterial use. The PPV for viral infection was lower: 75% for the protein panel and 87% for the mRNA panel. We note, however, that tests to positively identify viral infection are not currently available. As such, these tests, particularly the mRNA strategy, address an important diagnostic gap.

One study limitation includes the use of LOOCV rather than using independent training and validation cohorts. This was necessary since the protein panel demonstrated poor calibration using previously published model parameters. Although this may overestimate performance as compared to a truly independent validation, it enabled a head-to-head comparison between biomarker strategies. Another limitation is that we measured the protein panel using standardized research assays and not the proprietary ImmunoXpert™ platform. That could affect results, although we optimized the panel’s performance in this study cohort, which should mitigate technical differences. Most subjects in this study were adults. If distinguishing bacterial and viral etiologies in children is easier, then the differences observed between the three host response approaches may diminish. Children and adults may have distinct biological responses to infection. However, studies have shown that procalcitonin, the protein panel, and the mRNA panel evaluated here perform similarly in children and adults [12,18,21,40,52].Three of the four enrollment sites were in the same geographic area. To ensure generalizability of these results, further investigation must be done in a more heterogeneous population. We note that the indication for use for procalcitonin focuses on lower respiratory tract infection and therefore, its application to cases of upper respiratory tract ARI including pharyngitis may be more limited. Nevertheless, inclusion of a heterogeneous cohort strengthens the study’s generalizability. Lastly, all tests were performed on banked samples although the biomarkers evaluated here are not known to be affected by a single freeze-thaw cycle.

This study demonstrated a clear improvement in discriminating ARI etiologies using a gene expression approach, although it is unknown whether this will translate into improved patient management. Clinical trials are currently underway to investigate all three biomarker strategies and how best to apply the test results. Host response-based diagnostics offer new opportunities to characterize patients in ways not previously possible. Gene expression signatures, coupled with technical advances that bring these tests to clinical practice, potentially offer the best approach for host response-based infectious disease diagnosis.

## Supporting information

S1 Checklist STROBE statement checklist for cohort studies. (DOC)

S1 Table Adjudicated phenotype and biomarker measurements for each subject. (XLSX)
